# Perspectives into the experience of successful, substantial long-term weight-loss maintenance: a systematic review

**DOI:** 10.1080/17482631.2020.1862481

**Published:** 2021-01-17

**Authors:** Marie Spreckley, Jaap Seidell, Jutka Halberstadt

**Affiliations:** Faculty of Science, Department of Health Sciences, Vrije Universiteit Amsterdam, Amsterdam, Netherlands

**Keywords:** Sustainable weight loss, long-term weight loss, obesity management, weight management, weight loss

## Abstract

**Purpose**: In light of the increasing prevalence of overweight and obesity, understanding the experiences, strategies and challenges encountered when trying to achieve substantial, sustainable weight loss is an important area to investigate. We systematically evaluated qualitative studies focusing on the accounts of individuals who have achieved sustained weight loss to create a comprehensive picture of the experience of sustainable weight loss.

**Methods**: Included studies were peer-reviewed studies that qualitatively assessed the views and experiences of adults who previously had or currently have overweight or obesity who successfully lost weight and who subsequently maintained or regained weight. The evidence was systematically synthesized, which enabled the formulation of clear themes and recommendations.

**Results**: The 15 chosen studies included the accounts of 294 individuals. We found that continuous monitoring and goal setting, driven by sustained motivation and encouraging experiences, while resisting ever present challenges and enduring discouraging experience encapsulates the experience of sustained, substantial weight loss.

**Conclusions**: This review aims to provide a comprehensive understanding of the experiences, strategies and challenges encountered when trying to achieve substantial, sustained weight loss. Additional research taking into account findings from this review and others of its kind will enhance the formulation of treatment protocols.

## Section 1

### Background

Global obesity rates have increased nearly threefold over the past four decades (World Health Organisation, 2020). Notably, countries with the highest income disparity also have the highest rates of obesity. According to the WHO, over 1.9 billion adults over the age of 18 have overweight (26%) and 650 million have obesity (13%). It is important to note that, in the majority of countries, obesity now causes more mortalities than underweight, and the prevalence of overweight and obesity is increasing in developing countries as well (World Health Organisation, 2020). Childhood overweight and obesity are also continuously increasing globally, reaching over 340 million in 2018 (18%) (World Health Organisation, 2020). A global trend analysis investigated the body mass index (BMI) trends of 19.2 million adults from 200 countries between 1975 and 2014 utilizing 1698 measurement studies estimated that, based on current global trends, 18% of men and 21% of women will have obesity and 6% of men and 9% of women will have severe obesity by 2025 (NCD Risk Factor Collaboration (NCD-RisC), 2016).

In the UK, as in most developed countries, obesity rates are strongly correlated with social and economic circumstances, irrespective of age and gender (The Marmot Review, 2010). Although the cause of overweight and obesity may appear to be a simple imbalance in energy consumption compared to energy utilization, the underlying factors are vast, complex and varied. The Foresight Programme developed a complex web of interrelated, correlated and causative factors contributing to the increased prevalence of obesity including economic, social, medical, biological, developmental, infrastructural and psychological, among other factors (Foresight, 2007a).

In order to diagnose overweight and obesity, a variety of tools can be utilized in conjunction. The most common, primary measurement is the BMI, which is derived by dividing an adult’s weight by his/her squared height (NHS, 2019). Notably, individually differing body composition needs to be taken into account in addition to BMI measurements as muscle tissue, for example, can distort readings. Furthermore, ethnicity also needs to be taken into account as individuals from Asian origins, for instance, can have elevated health risks with a proportionally lower BMI (Royal College of Nursing, 2019). Thus, waist circumference (WC) is another valuable tool to assess risk. WC is deemed to be one of the most reliable measures to assess central obesity (Center for Disease Control, 2020). WC is particularly useful if a patient has a healthy BMI yet presents with significant abdominal adiposity (World Health Organisation, 2007).

Overweight and obesity increase the likelihood of developing a vast array of co-morbidities and can also significantly impact health-related quality of life. Co-morbidities conclusively associated with excess adiposity include cardiovascular disease, hypertension, hypercholesterolemia, type 2 diabetes, gastrointestinal complications, infertility, sleep apnoea, osteoarthritis (Pi-Sunyer, 2009) as well as 13 types of cancers (Cancer Research UK, 2020). Cancers conclusively linked to overweight and obesity include breast cancer, bowel cancer, gallbladder cancer, kidney cancer, liver cancer, meningioma, myeloma, oesophageal cancer, ovarian cancer, pancreatic cancer, stomach cancer, thyroid cancer and uterine cancer (Cancer Research UK, 2020).

In addition, overweight and obesity can impair quality of life in a multitude of ways. Individuals can feel isolated, stigmatized and excluded from participating in society. They also have an increased risk of developing anxiety and depression, which can lead to feelings of shame and vulnerability (Emmer et al., 2019). In addition, research has found that individuals with overweight and obesity are less likely to exercise publicly due to weight discrimination stigma (Foresight, 2007b). Thus, the health implications of overweight and obesity are multifaceted and need to be addressed from a multitude of angles.

A variety of dietary, physical activity, behavioural, pharmaceutical and surgical treatments are currently available for the treatment of overweight and obesity. Notably, practitioners are encouraged to work with individuals to both inform them about the benefits of weight loss as well as help determine which form of treatment is suitable for them (Fitzpatrick et al., 2016). Working in conjunction with individuals to determine which approach will yield sustainable adherence based on the patient’s preferences has shown to be the most beneficial treatment approach in the long run (Fitzpatrick et al., 2016). Diet, exercise and behavioural interventions are particularly useful tools for weight loss when used in conjunction (Adegboye & Linne, 2013; Franz et al., 2007; Wadden et al., 2015).

Research has shown that short-term weight loss utilizing a variety of approaches can be successful, yet more than 80% of successful individuals experience weight regain after 1 year, 85% after 2 years and over 95% after 3 years (Langeveld & de Vries, 2013). It is noteworthy that the majority of weight regainers gain more weight following successful short-term weight loss than they lost while dieting (Dulloo et al., 2012). Conversely, successful weight loss maintainers who have managed to maintain their weight loss for over 2 years are also more likely to maintain their weight loss over the subsequent 5 to 10 years (Natvik et al., 2018). Successful weight loss maintenance is frequently defined as 10% of intentional weight loss maintained over at least 1 year (Wing & Phelan, 2005), yet definitions vary and some also argue that 5% of intentional weight loss maintained over at least one year leads to clinically significant metabolic improvements and should therefore be seen as successful (Montesi et al., 2016). Intentional weight loss of at least 10% maintained over at least one year can therefore be considered successful and intentional weight loss of at least 5% maintained over at least one year can be considered moderately successful.

Although long-term adherence rates in weight loss studies remain suboptimal there is little focus on the individuals’ experience during weight loss interventions and the causes that lead to non-adherence during and after interventions. Thus, this is an important area to investigate further. Notably, individuals who experienced weight loss still had improved metabolic markers even following eventual weight regain, highlighting this important legacy effect of short and medium-term weight loss (Diabetes Prevention Programme (DPP) Research Group, 2002b, Diabetes Prevention Programme (DPP) Research Group, 2012, Hamdy et al., 2017; The Look AHEAD Research Group, 2013; Ravussin et al., 2015; Sacks et al., 2009), yet this legacy effect was not confirmed consistently across studies (Long et al., 2020).

Two notable systematic reviews conducted by Greaves et al. (2017) and Hartmann-Boyce et al. (2019) have investigated the experience of successful, substantial long-term weight loss maintenance. Greaves et al. found that a combination of continuous self-regulation, enduring motivation and the active management of external challenges supported by a changed self-concept as well as habit and need fulfilment alterations were the potential key to managing the tensions encountered when trying to achieve weight loss maintenance (WLM) (Greaves et al., 2017). Jamie Hartmann-Boyce et al. were very focused on the critical role of self-monitoring for WLM and found that success was frequently linked to strong self-knowledge and self-accountability, active, continuous self-monitoring resulting in actions, when required, and an underlying trust in both the approach and measures (Hartmann-Boyce et al., 2019). This review aims to contribute to this body of research. Additional research focusing on the experience of weight loss and weight loss maintenance will provide valuable insights into the challenges encountered as well as tools utilized to overcome barriers to success. This has the potential to enhance both personal and treatment success as it enhances the ability to create personalized weight loss strategies and treatment protocols.

### Aim and objective

The aim of this review is to systematically evaluate and analyse existing, qualitative studies investigating the experiences, challenges and strategies utilized to achieve long-term, substantial WLM.

The objective is to create an exploration of the experience of long-term WLM from the perspective of participants to provide deeper insights into the reality of the challenges encountered and strategies utilized.

This review will enable the formulation of evidence-based recommendations for long-term, substantial weight loss maintenance to aid in the management of overweight and obesity. It will also serve as the foundation for three consecutive, upcoming qualitative studies with a patient cohort from a primary care led weight loss programme in the UK to determine the impact the discovered themes can have on the achievement of successful, substantial WLM.

## Section 2

### Methods

#### Protocol and registration

This study is a systematic review and thematic synthesis of qualitative studies of long-term weight loss maintenance. Qualitative studies were chosen as they provide unique insights into the lived experience and enhance the ability to understand individual perceptions and views. Upon commencement of this review, the methods of the analysis and eligibility criteria were specified. A protocol was registered on the Open Science Framework (OSF) with DOI Identifier: DOI 10.17605/OSF.IO/8U23A. This study follows the ENTREQ guidance for qualitative syntheses (Tong et al., 2012). Bias assessment was conducted by Marie Spreckley, Prof. Dr. Jaap Seidell and Dr. Jutka Halberstadt.

#### Eligibility criteria

The criteria for the Chosen studies were decided upon prior to the commencement of research for this review.

The inclusion criteria consisted of peer-reviewed studies published in English since 1990 that qualitatively assess the views and experiences of individuals who previously or currently have overweight or obesity (BMI >25 kg/m^2^) who have attempted to achieve long-term weight loss maintenance for a minimum of one year. Attempts were defined as an individual taking proactive steps towards trying to achieve long-term weight loss by either participating in a structured weight loss programme or engaging continuously in specific behaviours with the aim to achieve weight loss. The main focus of the studies are the views and experiences of intentional weight loss maintenance within a broader investigation of weight management.

Studies excluded were non-human studies, paediatric studies, studies on bariatric surgery, studies reported in other languages than English, unintentional weight loss studies, non-qualitative studies and studies without participants who were overweight or obese before they lost weight.

Included studies provided input for our framework of common experiences of individuals with overweight and obesity during weight loss maintenance. This facilitated the formulation of recommendations for treatment strategies to support healthcare professionals.

### Search

The main databases searched were Pubmed, CINAHL and PsycINFO using controlled vocabulary. These databases were chosen as they provide a broad range of published, peer-reviewed studies in this specific subject area. The dates included ranged from 1990 to the end of March 2020. The searches were “weight loss maintenance”, “behaviour”, “experience”, “strategy”, “qualitative study” and “cohort study”. The searches were limited to qualitative, English, human, adult studies. The primary search was conducted in February 2020 and updated in March 2020.

### Synthesis of results

**Figure 1.**
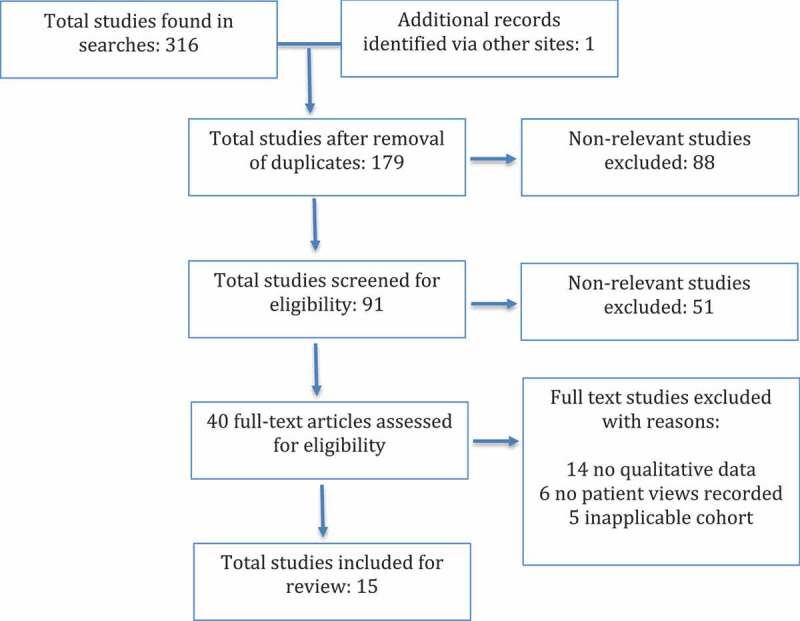
PRISMA flow diagram of studies identified

The eligible studies selected are summarized in the PRISMA flow diagram in Section 3 (Figure 1).

The focus was on assessing the confidence in the evidence, the confidence in the findings and the transferability and applicability for the present study (Lewin et al., 2019). The confidence in the studies was assessed by determining if there was a clear research question, study procedures were clearly described, there were clearly defined outcomes, the results support the conclusion and bias was unlikely (Table I).

**Table I.** Study quality assessments

Author (Year) Country	Validity Questions						Overall Quality Rating
	1	2	3	4	5	6	
Barnes et al. (2007) USA	Y	Y	Y	Y	Y	Y	Positive
Carrard and Kruseman (2016) Switzerland	Y	Y	Y	Y	Y	Y	Positive
Cleo et al. (2018) Australia	Y	N	Y	Y	Y	N	Neutral
Epiphaniou and Ogden (2010) UK	Y	Y	Y	Y	Y	N	Positive
Ingels and Zizzi (2018) USA	Y	N	Y	Y	Y	N	Neutral
Karfopoulou et al. (2013) Greece	Y	Y	Y	Y	Y	Y	Positive
Kruseman et al. (2017) Switzerland	Y	Y	Y	Y	Y	Y	Positive
Kwasnicka et al. (2019) Australia	Y	Y	Y	Y	Y	Y	Positive
McKee et al. (2013) UK	Y	Y	Y	Y	Y	Y	Positive
Metzgar et al. (2015) USA	Y	Y	Y	Y	Y	Y	Positive
Natvik et al. (2019) Norway	Y	Y	Y	Y	Y	Y	Positive
Natvik et al. (2018) Norway	Y	Y	Y	Y	Y	Y	Positive
Pedersen et al. (2018) Denmark	Y	Y	Y	Y	Y	Y	Positive
Reilly et al. (2015) Ireland	Y	Y	Y	Y	Y	Y	Positive
Sarlio-Lähteenkorva (2000) Finland	Y	Y	Y	Y	Y	Y	Positive
Y = Criteria met, N = Criteria not met							
**Validity Questions**	**Rating Method**						
1) Clear research question 2) Unbiased participant selection 3) Study procedure described 4) Clearly defined outcomes 5) Results support conclusions 6) Bias unlikely	Positive = Criteria 2,3,4, and 5 met Neutral = Criteria 2,3,4, or 5 not met Negative = Majority of criteria not met						

Y = Criteria met, N = Criteria not met

**Figure 2.**
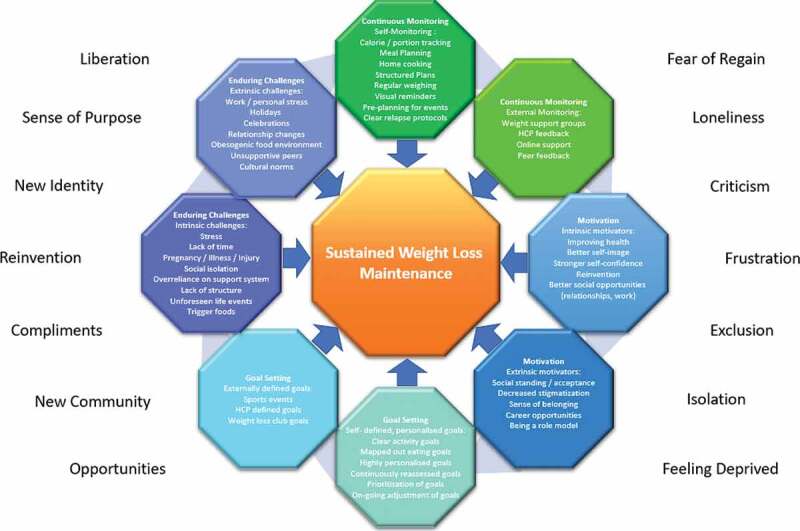
Determinants of sustainable weight loss—a framework

The approach for the synthesis of the findings for this paper has been adapted from the thematic synthesis approach for qualitative research developed by Thomas and Harden (2008). For this approach, the synthesis is subdivided into three consecutive steps. The first step consists of “free line-by-line coding”, which eventually leads to the second step, the collation of “descriptive themes”, which then leads to the last step, the development of the final themes (Thomas & Harden, 2008). The final themes remained descriptive in nature to ensure suitability for the developed of the framework (Figure 2).

Firstly, the key words were inserted into the databases as outlined in the “SEARCH” section. Once we found all titles in all databases, we collated them in an Excel spreadsheet with the correlated hyperlinks. In the spreadsheet, we simplified the title names to remove punctuation and grammatical differences to enable the removal of duplicates from multiple sources. Once this step was complete, we removed the duplicates and then filtered out titles that included terms from the exclusion criteria.

Next the abstracts of the remaining studies were read to exclude any studies that did not meet the inclusion criteria. This further narrowed the list. The remaining studies were read in full and unsuitable studies were removed. Once this step was complete, the findings of the identified studies were extracted and transferred for the next step, the line-by-line analysis.

The findings of the chosen studies were entered verbatim into a database and coded, line-by-line, according to their meaning and content. Some sentences were also assigned multiple codes, dependent on their content. This enables the translation of concepts between studies, resulting in a comprehensive synthesis of results across studies. The first author conducted the coding and the second and third author reviewed and revised the coding in collaboration with the first author. The descriptive themes that emerged upon initial review of the literature, which were used for the coding, were: (1) Motivation (green coding), (2) Self-Regulation/Monitoring (yellow coding), (3) Habits (purple coding), (4) Goal Setting (blue coding), (5) Challenges (grey coding), (6) Triggers (emerald coding) and (7) Environment/Support (olive coding).

After further collaborative analysis by all three authors, the final themes were developed to enable the formulation of a clear framework of experiences, challenges and strategies. This included the determination of influences, barriers, experiences, challenges as well as strategies utilized by participants to determine implications for intervention development. The 10 main themes that emerged were (1) continuous monitoring: self-monitoring (2) continuous monitoring: external monitoring (3) motivation: intrinsic motivators (4) motivation: extrinsic motivators (5) goal setting: self-defined (6) goal setting: externally defined (7) enduring challenges: intrinsic challenges, (8) enduring challenges: extrinsic challenges, (9) overall experience: encouraging experiences and (10) overall experience: discouraging experiences. The supporting data can be found in Table AI.

To determine the prevalence of each theme and subtheme in the literature, each theme and subtheme was transferred into a spreadsheet. Next, the supporting data from each study was added to the spreadsheet to be able to calculate the prevalence of each theme and subtheme across the 15 studies (Table II).

**Table II.** Hierarchy of prevalence

Frequency Mentioned/Prevalence	Theme	Category
Consistently Mentioned	Structured Plans Regular weighing Calorie/portion tracking Meal Planning Pre-planning for events Clear relapse protocols Visual reminders	Self-Monitoring
	Weight support groups Peer feedback HCP feedback	External Monitoring
	Better self-image Stronger self-confidence Improving health Better social opportunities (relationships, work)	Intrinsic Motivators
	Clear activity goals Mapped out eating goals Continuously reassessed goals Highly personalized goals Prioritization of goals	Self-Defined, Personalized Goals
	Unforeseen life events (death, divorce) Emotional eating	Intrinsic Challenges
	Holidays Celebrations Work/personal stress Unsupportive peers	Extrinsic Challenges
	New Community New Identity Reinvention	Encouraging Experiences
	Fear of Regain Criticism	Discouraging Experiences
Often Mentioned	Home cooking	Self-Monitoring
	Online support	External Monitoring
	Social standing/acceptance Decreased stigmatization	Extrinsic Motivators
	On-going adjustment of goals	Self-Defined, Personalized Goals
	HCP defined goals Weight loss club goals	Externally Defined Goals
	Stress Lack of time Cultural norms Pregnancy, Illness, Injury Trigger foods Overreliance on support system	Intrinsic Challenges
	Obesogenic Food Environment	Extrinsic Challenges
	Opportunities Compliments	Encouraging Experiences
	Frustration	Discouraging Experiences
Sometimes Mentioned	Being a role model Sense of belonging Career opportunities	Extrinsic Motivators
	Sports events	Externally Defined Goals
	Lack of structure Social isolation	Intrinsic Challenges
	Relationship changes	Extrinsic Challenges
	Feeling liberated Sense of purpose	Encouraging Experiences
	Isolation Exclusion Loneliness Feeling deprived	Discouraging Experiences

## Section 3

### Results

#### Study selection

The total number of suitable studies that met the inclusion criteria was 15 (Figure 1).

#### Screening

137 of the original 316 studies were found in more than one search. One additional study was found via Google. Once the duplicates were removed, 179 citations remained. These studies were screened for eligibility by reading the abstracts. Those not meeting the eligibility criteria were excluded. The reasons for exclusions included paediatric studies, bariatric surgery studies, drug/supplementation studies, unintentional weight loss studies, purely quantitative studies, review papers, studies not investigating the areas of interest and non-English studies. This resulted in 40 studies which, based on reading the abstracts, met the inclusion criteria for this review. The remaining studies were then screened in detail. Following this, a further 22 studies were excluded for the following reasons: 14 did not contain qualitative data, 6 did not record patient views and 5 did not meet the inclusion/exclusion cohort criteria. The reference sections were screened and berry picking was utilized, which led to the discovery of an additional, suitable study.

This search (Figure 1) produced a total of 15 studies that met the inclusion and exclusion criteria.

#### Selection process

##### Risk of bias within studies

This quality assessment tool was adapted from the American Dietetic Association quality assessment tool to determine suitability for this review (Academy of Nutrition and Dietetics, 2016).

#### Study characteristics

Overall, 294 female and male, adult, individuals of varying ethnicity participated in the identified studies, with sample sizes ranging from 9 to 44 participants. 32 of the 15 identified studies also included individuals who always maintained their weight within the healthy BMI range to date (Carrard & Kruseman, 2016; Kruseman et al., 2017; Reilly et al., 2015) and 5 had weight regainers (Barnes et al., 2007; Ingels & Zizzi, 2018; McKee et al., 2013; Pedersen et al., 2018; Reilly et al., 2015). This provides valuable, additional insights into what separates weight maintainers and regainers in terms of experiences, challenges and strategies utilized to achieve long-term weight loss maintenance.

The study protocols were also diverse consisting of focus groups (Barnes et al., 2007; Karfopoulou et al., 2013; Metzgar et al., 2015; Reilly et al., 2015), semi-structured interviews (Carrard & Kruseman, 2016; Cleo et al., 2018; Epiphaniou & Ogden, 2010; Ingels & Zizzi, 2018; Kwasnicka et al., 2019; McKee et al., 2013; Pedersen et al., 2018) open interviews (Natvik et al., 2018 , 2019; Reilly et al., 2015; Sarlio-Lähteenkorva, 2000) and the additional utilization of surveys (Kruseman et al., 2017). A summary of the characteristics can be found in Table III.

**Table III.** Summary of data extracted from studies

Author (Year); Country	Title	Aim	Sample Size	Approach	Weight Loss Maintenance Criteria
Barnes et al. (2007) USA	Weight Loss Maintenance in African–American Women: Focus Group Results and Questionnaire Development	To understand the experience of weight loss maintenance in African-American women	37	Qualitative study consisting of 7 focus groups, 4 with successful WLMs, 3 with regainers.	Two groups: 1) Maintainers = Intentional weight loss ≥10% of BW sustained ≥1 year. 2) Regainers = Intentional weight loss ≥10% of BW but regained the lost weight.
Carrard and Kruseman (2016) Switzerland	Qualitative Analysis of the Role of Self-Weighing as a Strategy of Weight Control for Weight-Loss Maintainers in Comparison with a Normal, Stable Weight Group	To understand the role of self-weighing for weight control utilized by WLMs compared to weight stable group.	18	Qualitative study consisting of individual, face-to-face, semi-structured interviews with all participants.	Two groups: 1) Intentional weight loss of at ≥10% sustained ≥1 year 2) Healthy BMI range during adulthood stable within a 5 kg range
Cleo et al. (2018) Australia	Participant Experiences of Two Successful Habit-Based Weight-Loss Interventions in Australia: A Qualitative Study	To understand the experiences and perspectives of participants from a series of 2-year, habit-based weight-management programme.	15	Qualitative study consisting of individual, face-to-face, semi-structured interviews with all participants.	Weight change ranging from −10.4% BW to +4.0% BW over 1 year.
Epiphaniou and Ogden (2010) UK	Successful Weight Loss Maintenance and a Shift in Identity from Restriction to a New Liberated Self	To understand the experiences of weight loss for successful WLMs with a focus on self- perception from their heaviest to their current weight.	10	Qualitative study consisting of individual, face-to-face and phone semi-structured interviews with all participants.	Intentional weight loss ≥10% of BW sustained ≥1 year.
Ingels and Zizzi (2018) USA	A Qualitative Analysis of the Role of Emotions in Different Patterns of Long-Term Weight Loss	To understand and analyse the emotions entailed in different patterns of long-term weight loss	21	Qualitative study consisting of semi-structured interviews with weight management programme participants.	Three groups over 18 months: 1) large loss = intentional weight loss of ≥7% of BW 2) moderate loss = intentional weight loss of ≥3–6% of BW 3) regainer = no loss or weight gain
Karfopoulou et al. (2013) Greece	Behaviours Associated With Weight Loss Maintenance and Regaining in a Mediterranean Population Sample. A Qualitative Study	To understand lifestyle behaviours associated with weight loss and WLM in participants who had lost ≥10% of BW and either kept if off for ≥1 year or regained it.	44	Qualitative study consisting of 8 focus groups, 4 with WLMs and 4 with regainers, focusing on the experience and beliefs around weight loss and WLM.	Intentional weight loss ≥ 10% of BW sustained ≥1 year.
Kruseman et al. (2017) Switzerland	Long-Term Weight Maintenance Strategies Are Experienced as a Burden by Persons Who Have Lost Weight Compared to Persons with a lifetime Normal, Stable Weight	To understand strategies and perceptions of the experience of WLMs in participants who have lost ≥10% of BW and kept if off for ≥1 year compared with matched, lifelong normal weight controls.	32	Mixed methods, cross-sectional study consisting of surveys and semi-structured interviews. Interviews investigated strategies and experiences of WLM.	Intentional weight loss ≥10% of BW sustained ≥1 year.
Kwasnicka et al. (2019) Australia	‘It’s not a diet, it’s a lifestyle’: A Longitudinal, Data-Prompted Interview Study of Weight Loss Maintenance	To understand the factors underpinning WLM (individual and environmental).	12	Qualitative study consisting of semi-structured, data-prompted interviews with 12 participants that had lost ≥5% of body weight (BW) in the year prior.	Intentional weight loss of ≥5% of BW sustained ≥1 year.
McKee et al. (2013) UK	Weight Maintenance: Self-Regulatory Factors Underpinning Success and Failure	To understand the contributing factors involved in weight maintenance both success and failure	18	Qualitative study consisting of semi-structured interviews with successful and unsuccessful WLMs.	Two groups: 1) Maintainers = Intentional weight loss ≥10% of BW sustained ≥1 year 2) Regainers = Previously achieved intentional weight loss ≥10% of BW yet unable to maintain the new weight for a 1 year.
Metzgar et al. (2015) USA	Facilitators and Barriers to Weight Loss and Weight Loss Maintenance: A Qualitative Exploration	To understand facilitators and barriers to weight loss and WLM in individuals who participated in primary care weight loss study.	23	Qualitative study consisting of 7 focus groups using open-ended questions and probes with WLMs and regainers.	Intentional weight lost ≥13.6 kg sustained for ≥1 year.
Natvik et al. (2019) Norway	An Experientially Derived Model of Flexible and Intentional Actions for Weight Loss Maintenance After Severe Obesity	To understand the experiences of weight loss and WLM for ≥5 years following severe obesity.	10	Qualitative study consisting of individual, open, in-depth interviews focused on the experience of weight loss and WLM after severe obesity.	Intentional weight loss of ≥10% of BW sustained ≥5 years.
Natvik et al. (2018) Norway	Living a Successful Weight Loss After Severe Obesity	To understand the experiences of successful weight loss after severe obesity.	10	Qualitative study consisting of individual, in-depth, face to-face interviews with WLMs who have maintained a 10% weight loss for ≥5 years.	Intentional weight lost over 10% of BW sustained ≥5 years.
Pedersen et al. (2018) Denmark	The Complexity of Self-Regulating Food Intake in Weight Loss Maintenance. A Qualitative Study Among Short- and Long-Term Weight Loss Maintainers	To understand self-regulatory strategies and self-eﬃcacy beliefs utilized by short- and long-term WLMs.	18	Qualitative study consisting of individual, semi-structured interviews with 9 short and 9 long-term WLMs focusing on self-regulatory strategies and self-eﬃcacy beliefs.	Two groups with intentional weight loss ≥10% of BW with no more than 2 kg regain and a stable weight for at ≥2 months. 1) Short-term maintainers (2–12 months) 2) Long-term maintainers (≥12 months)
Reilly et al. (2015) Ireland	Lessons Learned about Primary Weight Maintenance and Secondary Weight Maintenance: Results From a Qualitative Study	To understand attitudes, behaviours, motivations and strategies of WLMs, lifelong weight maintainers and weight regainers.	17	Qualitative study consisting of 7 focus groups and 3 individual interviews with primary and secondary weight maintainers and weight regainers.	Three groups: 1) Participants who maintained their weight within a healthy BMI range over their lifetime 2) Participants who achieved intentional weight loss ≥6.35 kg sustained ≥1 year. 3) Participants who tried repeatedly but had not lost weight in the past year
Sarlio-Lähteenkorva (2000) Finland	‘The battle is not over after weight loss’: Stories of Successful Weight Loss Maintenance	To understand the experience of weight loss maintenance among the ‘reduced-obese’.	9	Qualitative study consisting of open interviews with nine women who lost 10–27 kg of weight and kept it off for ≥7 years.	Intentional weight loss of ≥10 kg sustained ≥7 years.

The criteria used for successful weight loss maintenance differed significantly across the identified studies. Seven studies used the above definition of having achieved ≥10% of body weight loss sustained over 1 year (Barnes et al., 2007; Carrard & Kruseman, 2016; Epiphaniou & Ogden, 2010; Karfopoulou et al., 2013; Kruseman et al., 2017; McKee et al., 2013; Pedersen et al., 2018), while others defined it as having achieved ≥10% of body weight loss sustained over 5 years (Natvik et al., 2019, 2018). Some defined it as having achieved ≥10 kg of body weight loss sustained over 7 years (Sarlio-Lähteenkorva, 2000) and others defined it as having achieved ≥5% of body weight loss sustained over 1 year (Kwasnicka et al., 2019), having achieved ≥13.6 kg of body weight loss sustained over 1 year (Metzgar et al., 2015), having achieved ≥6.35 kg of body weight loss sustained over 1 year (Reilly et al., 2015) and having achieved ≥7% of body weight loss sustained over 1 year (Ingels & Zizzi, 2018). One study included participants whose weight change ranged from having achieved a 10.4% body weight loss to having regained up to 4.0% of body weight over a year (Cleo et al., 2018). A summary of the criteria for weight loss maintenance can be found in Table III.

#### Study findings

##### Continuous monitoring

The theme of continuous monitoring was most consistently mentioned throughout all studies. This was divided into both self-monitoring and external monitoring and participants consistently stated that having clear monitoring tools in place for both weight loss as well as WLM were what kept them on-track, aware and accountable.

###### Self-monitoring

Successful WLMs discussed a variety of self-monitoring tools. Particularly calorie and portion control featured heavily as both gave participants a way to track how much they were eating and balance out their intake after overindulging (Karfopoulou et al., 2013; Kwasnicka et al., 2019; McKee et al., 2013; Natvik et al., 2018, 2019; Reilly et al., 2015; Sarlio-Lähteenkorva, 2000). Tools utilized included calorie-tracking apps (Natvik et al., 2019; Reilly et al., 2015), food diaries (McKee et al., 2013; Natvik et al., 2019) and food scales (Natvik et al., 2019; Reilly et al., 2015). Another important tool was remaining mindful about portion sizes (Karfopoulou et al., 2013; Kwasnicka et al., 2019; McKee et al., 2013; Natvik et al., 2018, 2019; Reilly et al., 2015; Sarlio-Lähteenkorva, 2000). Meal planning was also mentioned as a crucial component including planning and preparing meals in advance for regular consumption to stay on track and remain in control during challenging situations (Carrard & Kruseman, 2016; Karfopoulou et al., 2013; Kwasnicka et al., 2019; Pedersen et al., 2018; Reilly et al., 2015; Sarlio-Lähteenkorva, 2000). Successful WLMs also frequently pre-planned for events, utilizing strategies like pre-preparing food to consume at events (Carrard & Kruseman, 2016; McKee et al., 2013; Metzgar et al., 2015; Reilly et al., 2015) and checking menus and food choices to plan what to eat in advance (Carrard & Kruseman, 2016; Metzgar et al., 2015; Natvik et al., 2019; Pedersen et al., 2018; Sarlio-Lähteenkorva, 2000). Home cooking was often employed as a strategy to both learn about ingredients and prepare the appropriate portions to stay on track (Carrard & Kruseman, 2016; Kwasnicka et al., 2019; Pedersen et al., 2018).

Having clear, structured plans for how WLM will be maintained featured heavily in the studies (Barnes et al., 2007; Carrard & Kruseman, 2016; Karfopoulou et al., 2013; Kruseman et al., 2017; Kwasnicka et al., 2019; Metzgar et al., 2015; Natvik et al., 2018; Pedersen et al., 2018; Reilly et al., 2015; Sarlio-Lähteenkorva, 2000). WLMs relied on clear routines for their everyday lives (Carrard & Kruseman, 2016; Kruseman et al., 2017; Kwasnicka et al., 2019; Metzgar et al., 2015; Natvik et al., 2018; Pedersen et al., 2018; Reilly et al., 2015; Sarlio-Lähteenkorva, 2000), clear eating times (Karfopoulou et al., 2013; Kwasnicka et al., 2019; Pedersen et al., 2018) as well as limiting food choices (Carrard & Kruseman, 2016; Karfopoulou et al., 2013; Kruseman et al., 2017; Metzgar et al., 2015; Natvik et al., 2018; Pedersen et al., 2018; Reilly et al., 2015; Sarlio-Lähteenkorva, 2000).WLMs utilized regular weighing as a self-monitoring tool as it provided them with a sense of remaining on track and in control throughout their journeys (Carrard & Kruseman, 2016; Epiphaniou & Ogden, 2010; Karfopoulou et al., 2013; Kwasnicka et al., 2019; McKee et al., 2013; Natvik et al., 2018, 2019; Reilly et al., 2015; Sarlio-Lähteenkorva, 2000). Visual reminders including progress photos served as motivators and cautionary reminders to stay focused (Epiphaniou & Ogden, 2010; Natvik et al., 2019; Pedersen et al., 2018; Sarlio-Lähteenkorva, 2000). The importance of having clear relapse protocols was also highlighted, including approaches like acceptable weight ranges (Carrard & Kruseman, 2016; Karfopoulou et al., 2013; Natvik et al., 2019; Sarlio-Lähteenkorva, 2000) and how clothes fit (Barnes et al., 2007; Karfopoulou et al., 2013).

###### External monitoring

The role of external monitoring was also significant for WLM and served as a facilitator for continuous self-monitoring. Weight support groups provided a space to have accountability (Metzgar et al., 2015), support and motivation (Metzgar et al., 2015; Reilly et al., 2015; Sarlio-Lähteenkorva, 2000). Participants also appreciated guidance, interest and support from their HCPs during and after their weight loss journey (Cleo et al., 2018; Metzgar et al., 2015; Natvik et al., 2018, 2019). Having online support was perceived as an additional motivator by some participants (Cleo et al., 2018; Natvik et al., 2019). Feedback from friends, family, work colleagues and peers served as a significant motivator to stay on track and enhanced motivation for participants across studies to stay on track (Kwasnicka et al., 2019; Metzgar et al., 2015; Natvik et al., 2018, 2019; Reilly et al., 2015; Sarlio-Lähteenkorva, 2000).

##### Motivation

Intrinsic and extrinsic motivators were the drivers that encouraged individuals to stay on track and remain motivated to achieve sustained WLM. Participants were particularly encouraged by intrinsic motivators, yet extrinsic motivators also played a critical role.

###### Intrinsic motivators

Intrinsic motivators included the desire to improve their health, ranging from individuals wishing to improve their overall quality of life and fitness (Barnes et al., 2007; Epiphaniou & Ogden, 2010; Karfopoulou et al., 2013; Kwasnicka et al., 2019; Natvik et al., 2019; Reilly et al., 2015; Sarlio-Lähteenkorva, 2000) to trying to improve or prevent specific, weight-related conditions (Karfopoulou et al., 2013; Natvik et al., 2019; Reilly et al., 2015; Sarlio-Lähteenkorva, 2000). WLMs also described that their self-image improved significantly due to their weight loss and felt that they had regained control of their life and developed a positive self-image (Barnes et al., 2007; Cleo et al., 2018; Epiphaniou & Ogden, 2010; Karfopoulou et al., 2013; Natvik et al., 2018, 2019; Pedersen et al., 2018; Sarlio-Lähteenkorva, 2000). This new reality also enhanced their self-esteem in multiple areas of their lives including how they felt about themselves and how they approached relationships (Cleo et al., 2018; Epiphaniou & Ogden, 2010; Karfopoulou et al., 2013; Kruseman et al., 2017; Natvik et al., 2018, 2019; Reilly et al., 2015). Participants spoke of a sense of “reinvention” and an identity shift to a new lifestyle after successful weight loss (Epiphaniou & Ogden, 2010; Kwasnicka et al., 2019; Natvik et al., 2018, 2019). Better social opportunities were frequently cited as motivators in multiple areas including personal relationships and work, often facilitated by their new found self-esteem and self-image (Epiphaniou & Ogden, 2010; Karfopoulou et al., 2013; Natvik et al., 2018, 2019; Reilly et al., 2015; Sarlio-Lähteenkorva, 2000).

###### Extrinsic motivators

Extrinsic motivators were more diverse, with some feeling motivated to enhance their social standing and acceptance by becoming mentors for others trying to lose weight and re-educating themselves to do so (Natvik et al., 2019). Others were driven by the wish to have a more socially acceptable body shape, which helped them improve their confidence when interacting with others (Epiphaniou & Ogden, 2010). The wish to decrease social stigmatization was also prevalent as participants had experienced this in various settings and to varying degrees prior to their weight loss (Epiphaniou & Ogden, 2010; Karfopoulou et al., 2013). Support systems also gave participants a sense of belonging, which motivated them to stay on track (Kwasnicka et al., 2019). Others found that their weight loss provided them with enhanced career opportunities and pursued further education to facilitate this (Natvik et al., 2019). Some were motivated to become role models for others struggling with their weight by joining networks and participating in high-level sports competitions (Natvik et al., 2019).

##### Goal setting

Clear, personalized, continuously adjusted goals were an important theme consistently present throughout the studies. Self-defined, personalized goals appeared more significant than externally defined goals yet were frequently guided by external input.

###### Self-defined, personalized goals

Having clear activity goals stood out as WLMs had regular gym schedules (Kwasnicka et al., 2019) or engaged in regular sports events (Kruseman et al., 2017; Natvik et al., 2019, 2018; Sarlio-Lähteenkorva, 2000). WLMs were generally more physically active in their daily lives than before (Barnes et al., 2007; Karfopoulou et al., 2013; Kruseman et al., 2017; Kwasnicka et al., 2019; McKee et al., 2013; Reilly et al., 2015; Sarlio-Lähteenkorva, 2000). Consistently mapped out eating goals were frequently mentioned ranging from overall food consumption goals (Kwasnicka et al., 2019; Natvik et al., 2019) to food category and macronutrient-specific goals (Kruseman et al., 2017). Having highly personalized goals that could be adjusted according to expected and unexpected life events seemed to enhance WLM autonomy and success (Reilly et al., 2015; Sarlio-Lähteenkorva, 2000). WLMs who continuously reassessed if their goals were still suitable felt that this enhanced their continuous commitment to WLM (Natvik et al., 2019; Sarlio-Lähteenkorva, 2000). A continuous effort to prioritize weight loss maintenance over possible derailing circumstances (Kruseman et al., 2017; McKee et al., 2013; Natvik et al., 2018; Sarlio-Lähteenkorva, 2000) along with an ongoing adjustment of goals to solidify a lifelong commitment to change provided a strong foundation for successful WLM (Natvik et al., 2019, 2018).

###### Externally defined goals

Externally defined goals provided guidance, accountability and support, ranging from sports events (Natvik et al., 2019, 2018) to HCP defined goals (Cleo et al., 2018; Natvik et al., 2019) to weight loss club goals (Sarlio-Lähteenkorva, 2000). Notably, in this particular selection of studies, externally defined goals appeared to be beneficial yet not essential driving factors.

##### Enduring challenges

WLMs were frequently faced with challenges and found enduring them one of the hardest aspects of sustained WLM. Challenges were both intrinsic and extrinsic in nature and threatened WLM on an ongoing basis.

###### Intrinsic challenges

Intrinsic challenges ranged from everyday stress (Karfopoulou et al., 2013; Kwasnicka et al., 2019; Pedersen et al., 2018) to a lack of time to engage in WLM associated behaviours like exercise (Kwasnicka et al., 2019; Metzgar et al., 2015). Emotional eating was another challenge that featured strongly in the studies, which was frequently utilized to regulate emotions as well as manage stress and boredom (Epiphaniou & Ogden, 2010; Ingels & Zizzi, 2018; Karfopoulou et al., 2013; Reilly et al., 2015). Life events such as pregnancy, illness or injury also challenged WLM success, potentially interrupting important routines and habits to maintain weight loss success (Reilly et al., 2015). Some also felt socially isolated and that their WLM was a burden to their peers (Kruseman et al., 2017), while others developed an overreliance on their WLM support system, often to regulate their emotions and keep them motivated (Ingels & Zizzi, 2018; McKee et al., 2013). A lack of structure also led to participants struggling to manage their food consumption patterns long term (Reilly et al., 2015). Major life events including deaths, divorce, job losses and changes in living arrangements posed ongoing threats to success that actively needed to be managed (Karfopoulou et al., 2013; Reilly et al., 2015). Trigger foods and temptations, particularly during challenging times, were omnipresent and continuously threatened WLM success (McKee et al., 2013; Sarlio-Lähteenkorva, 2000).

###### Extrinsic challenges

Work and personal stress due to external influences including stressful meetings, house moves or relationship break-ups, amongst others, were potential threats to sustained WLM that needed to be managed on an ongoing basis (Barnes et al., 2007; Kwasnicka et al. 2019; Reilly et al., 2015). Holidays were another regular occurrence that made sticking to WLM routines and habits challenging as these were frequently tied to home environments and relied on access to home resources (Carrard & Kruseman, 2016; Kwasnicka et al., 2019; Natvik et al., 2018). Celebrations such as birthday parties, weddings and other celebratory occasions were challenging to navigate as they were frequent occurrences that needed to be managed mindfully (Carrard & Kruseman, 2016; Natvik et al., 2018; Sarlio-Lähteenkorva, 2000). Relationship changes also had an impact on everyday routines that required restructuring and adjustment as well as emotional stamina for WLM (Karfopoulou et al., 2013). Obesogenic food environments felt daunting due to the ever presence of hyperpalatable, hypercaloric, inexpensive foods threatening to derail WLM on a continuous basis (Kwasnicka et al., 2019; Natvik et al., 2018). New routines and increased self-confidence due to weight loss and WLM was often met by unsupportive peers who would discourage, tempt and pressure WLM to engage in counterproductive activities like overeating and drinking excessively (Kwasnicka et al., 2019; Metzgar et al., 2015; Pedersen et al., 2018; Sarlio-Lähteenkorva, 2000). Cultural norms concerning both portions and kinds of food as well as cultural norms around alcohol consumption were often challenging areas to navigate for WLMs (Barnes et al., 2007; Kwasnicka et al., 2019).

##### Overall experience

The overall experience of WLM entailed both very encouraging and discouraging elements. Depending on the individual, their surroundings and the context, positive experiences could be life changing, while negative experiences could be disheartening.

###### Encouraging experiences

WLMs expressed feeling liberated, like a new person who is free from weight concerns (Epiphaniou & Ogden, 2010). They described having found a new sense of purpose by channelling their energy into educating themselves and like-minded peers in various areas including dieting and exercise to help them attain what they had achieved (Natvik et al., 2019). Participants spoke of having developed new identities, transforming into people who lived healthy lives, took chances, pursued previously daunting opportunities and felt they had existentially changed indefinitely (Epiphaniou & Ogden, 2010; McKee et al., 2013; Natvik et al., 2018, 2019). Participants spoke of having reinvented themselves by fundamentally shifting their identity, speaking of a second chance at life as this reinvented version of themselves (Epiphaniou & Ogden, 2010; Kwasnicka et al., 2019; Natvik et al., 2018). WLMs also enjoyed receiving compliments and admiration for their success (Natvik et al., 2019, 2018) and appreciated being included in new communities that they were either too insecure to approach or had felt excluded from previously (Kwasnicka et al., 2019; Natvik et al., 2019, 2018; Sarlio-Lähteenkorva, 2000). Opportunities priorly inaccessible both socially, e.g., having children, physically, e.g., competing in sports, and at work had become accessible following WLM, which encouraged WLMs to stay on course continuously (Epiphaniou & Ogden, 2010; Natvik et al., 2019).

###### Discouraging experiences

Conversely, the fear of regain was ever-present for a significant number of WLMs, making the journey of WLM feel like an ongoing challenge and burden that needs to be continuously combatted (Kruseman et al., 2017; Natvik et al., 2018; Reilly et al., 2015; Sarlio-Lähteenkorva, 2000). WLM was perceived as a lonely process at times (Kruseman et al., 2017) and participants felt frequently criticized for their choices and lifestyle by their peers (Metzgar et al., 2015; Pedersen et al., 2018; Sarlio-Lähteenkorva, 2000). Some WLMs also felt frustrated and fatigued with the effort and challenges entailed in WLM (Karfopoulou et al., 2013; Reilly et al., 2015) and felt isolated when trying to limit their consumption patterns in groups (Pedersen et al., 2018). Being unable to resume previous consumption patterns after achieving their weight loss goals also left some participants feeling deprived (Karfopoulou et al., 2013).

##### Lessons learned from weight regainers

Five studies included accounts of participants who did not manage to achieve sustainable weight loss (Barnes et al., 2007; Ingels & Zizzi, 2018; McKee et al., 2013; Pedersen et al., 2018; Reilly et al., 2015). Weight regainers spoke of finding it hard to prioritize tracking their intake and dedicating time to exercise (Barnes et al., 2007; Ingels & Zizzi, 2018; Reilly et al., 2015). They frequently cited turning to emotional eating during stressful situations or due to boredom and using food as a coping mechanism (Ingels & Zizzi, 2018; Reilly et al., 2015) and reward (Reilly et al., 2015). They also highlighted a lack of planning as counterproductive (Pedersen et al., 2018; Reilly et al., 2015) and found it challenging to try and resist urges and temptations (Barnes et al., 2007; Pedersen et al., 2018; Reilly et al., 2015). Some also found it hard to put their weight loss above the needs and wishes of their peers and gave in to social pressure (Barnes et al., 2007; Pedersen et al., 2018). Further barriers to WLM included not wanting to waste food and feeling deprived due to dietary over-restriction (Pedersen et al., 2018). Weight fluctuations and every day stressors also consistently decreased motivation (Barnes et al., 2007; Reilly et al., 2015). Participants felt that being unable to maintain their weight loss negatively impacted their self-esteem, sometimes leading to secret eating and binge eating (Reilly et al., 2015).

### Evidence synthesis

This figure illustrates the framework of themes encompassing the experience of sustained weight loss maintenance. The categories around the wheel correlate with the themes and sub-themes identified and the overall experience is illustrated on the right and left side of the wheel (Appendix—Table AI). HCPs can use this framework upon commencement of treatment to devise personalized treatment plans for their patients.

## Section 4

### Discussion

The aim of this review was to systematically analyse the experiences, challenges and strategies utilized by successful WLMs with the objective to gain comprehensive insights based on personal accounts. This has enabled us to develop a framework for sustainable weight loss maintenance as well as formulate recommendations to aid in the management of overweight and obesity. Themes that emerged frequently centred around the importance of continuous monitoring and personalized, continuously evolving goal setting. This was driven by sustained motivation, often fuelled by encouraging experiences, while resisting challenges and enduring potentially discouraging experiences.

### Comparison with other research

Studies investigating sustained WLM are moving away from focusing predominantly on the best dietary and exercise approaches to investigating the social determinants impacting sustained WLM. This shift is also shining a light on the underlying behavioural aspects underpinning WLM success, which rely on understanding the experiences, strategies and challenges individuals encounter. The themes of continuous self-monitoring supported by strong motivation and peer feedback, physical activity goals and clear relapse protocols are consistently mentioned throughout WLM research (Elfhag & Rossner, 2005; Garip & Yardley, 2011; Simpson et al., 2011; Teixeira et al., 2015; Wing & Phelan, 2005). Greaves et al. also found that self-monitoring, enduring motivation and the continuous management of external challenges were important elements to achieve substantial, significant WLM (Greaves et al., 2017). Hartmann-Boyce et al. found that self-monitoring and an underlying trust in both the approach and accountability measures enhanced success (Hartmann-Boyce et al., 2019). The insights that emerged from this review are in line with these previous findings. The findings of this review also highlight important additional themes including the significance of continuously redefined, ever evolving, individualized goal setting, which has also been identified by Archibald et al. and Avery et al. (Archibald et al., 2015; Avery et al., 2016), and the impact encouraging experiences including a sense of purpose, liberation and reinvention have on significant, substantial WLM, as also determined by Ogden and Hills (2008).

The aim of developing this guiding framework for HCPs (Figure 2) was to provide a foundation for the formulation of strategies that enhance WLM. This framework entails elements in line the “Conceptual model of the dynamics of weight loss maintenance” by Greaves et al. including the intrinsic and extrinsic challenges encountered, the importance of self-monitoring and continuous motivation and the significant role of the self-concept and identity for WLM success (Greaves et al., 2017). The discoveries of this review also enabled the addition of notable elements for WLM success that have the potential to optimize treatment strategies further. Merging the already established elements with the additional findings will likely enhance outcomes. The additions include the importance of continuously evolving, personalized goal setting to stay on track and the role external support and monitoring can play to enhance success, which have both been found to be result enhancing (Archibald et al., 2015; Avery et al., 2016), as well as providing a holistic picture of the overall experience of WLM.

### Implications for research

Further research into the accounts of individuals who have achieved sustained WLM will enhance treatment quality and will lend scientific support for the creation and funding of treatment protocols. Additionally, a shift from retrospective research to longitudinal research utilizing frameworks to devise individualized treatment protocols upon commencement of treatment also provide an opportunity to investigate the benefits of having targeted approaches. These additional insights shine a light on areas that warrant further investigation and need to be considered to optimize long-term outcomes.

### Implications for practice

The findings of this review highlight the following aspects for HCPs. Helping individuals create clear, structured plans and an overall framework that is achievable and sustainable within their individual setting appears to be the foundation for achieving sustained WLM. Treatment strategies within that include providing tools that enable calorie and portion monitoring, recommending regular weighing and meal planning, using visual tools like progress pictures and reminders to stay motivated and having clear relapse protocols. Participants frequently mentioned appreciating HCP feedback and support as well as group and peer support so providing a clear, consistent feedback structure and a peer support system are advisable components for treatment programmes.

Participants also consistently noted that having clear, personalized activity and eating goals that were continuously prioritized, reassessed and adjusted helped them stay on track and feel in control. This was particularly useful when faced with extrinsic challenges to regular routines like holidays, celebrations, work and personal stress as well as being encouraged to overconsume by unsupportive peers, which was a frequent occurrence. Unforeseen life events like a death or divorce were also mentioned as potential threats to WLM as this frequently led to participants resorting to emotional eating as a coping mechanism. These elements will need to be taken into account when devising treatment protocols to enhance WLM.

It is equally beneficial for HCPs and individuals wishing to achieve WLM to understand the underlying dynamics of successful WLM, including that participants were strongly motivated by their desire to achieve WLM to improve their self-image, strengthen their self-confidence as well as gain better social opportunities, ranging from personal relationships to career opportunities. Having the chance to experience a reinvention of themselves within previously inaccessible communities and creating a new identity was seen as profoundly motivating. Participants spoke of having a second chance in life and were fearful of losing this opportunity. Thus, the fear of regain was ever present for a significant number of participants. Changing lifestyles and often communities also lead to criticism and sabotage from peers who had grown accustomed to the previous status quo, making some relationships and interactions unsustainable for WLMs. An awareness of these underlying dynamics has the potential to significantly enhance treatment and personal WLM success. HCPs can utilize the developed Framework (Figure 2) upon commencement of treatment to devise individualized treatment protocols. Particularly within the setting of a multidisciplinary team, patients could be referred to different treatment pathways upon commencement to enhance long-term WLM success. Individuals struggling with social isolation, for example, might have differing needs from someone surrounded by unsupportive peers.

### Strengths and limitations

The strengths of this review include the ability to gain a deeper understanding of the experience of WLM based on personal accounts. Personal accounts provide the opportunity to discover potentially unexplored experiences and elucidate the prevalence of commonly shared experiences. Limitations include the previously detailed inclusion and exclusion criteria as it restricted the comparability of the found datasets (defined in “Eligibility Criteria” on page 14) due to their variance. Paediatric studies were excluded as approaches and experiences differ significantly for children and adults. Unintentional weight loss studies, bariatric surgery studies and studies without participants who had/have overweight and obesity were excluded to ensure the main objective of this review can be fulfilled. The inability to generalize the findings of this study to children or people who had bariatric surgery is a noteworthy limitation. A further notable area is that participants may experience memory and recall bias. Emerging research suggests that participants in weight loss programmes retrospectively perceived periods of successful weight loss more positively and periods of regain more negatively than previously indicated (Ross & Wing, 2018). Therefore, the findings of this systematic review have to be seen in light of the above-mentioned limitations.

## Conclusions

This review highlights the importance of gaining a comprehensive understanding of the experiences, challenges and strategies that enhance significant, sustained WLM. Topics that consistently emerged were the need for clear, structured plans and frameworks supported by a network of peer and HCP support that take into account the fluidity of life and individual personalities and needs. The majority of WLMs viewed the experience of successful WLM as a chance to reinvent themselves as well as an opportunity to create a new identity and access new communities. However, this opportunity was often accompanied by a continuous fear of weight regain as well as criticism from unsupportive peers. Further research into the experiences, strategies and challenges of WLM, taking into account findings from this review as well and others of its kind, will contribute valuable insights into the experience of WLM, which will enhance the ability to formulate successful treatment protocols.

Final search strategy, 5 March 2020

### Search

The main databases searched were Pubmed, CINAHL and PsycINFO using controlled vocabulary. The dates included ranged from 1990 to the end of March 2020. The searches were “weight loss maintenance”, “behaviour”, “experience”, “strategy”, “qualitative study” and “cohort study”. The searches were limited to qualitative, English, human, adult studies. The primary search was conducted in February 2020 and updated in March 2020.

The searches were executed the following way:

#### Pubmed search

Pubmed was searched for applicable studies from 1990 to the end of March 2020 using the terms below in three separate searches (“weight loss maintenance’’, “experience”, “behaviour”, “strategy”, “qualitative study” and “cohort study”). The searches were limited to “human” and “English language”.

“weight loss maintenance” and “behaviour” identified 42 papers.

“weight loss maintenance” and “experience” identified 35 papers.

“weight loss maintenance” and “strategy” identified 35 papers.

“weight loss maintenance” and “qualitative study” identified 15 papers.

#### CINAHL search

CINAHL was searched from 1990 to the end of March 2020 using the terms below in three separate searches (“weight loss maintenance’’, “experience”, “behaviour”, “strategy”, “qualitative study” and “cohort study”). The searches were limited to “human” and “English language”.

“weight loss maintenance” and “behaviour” identified 27 papers.

“weight loss maintenance” and “experience” identified 14 papers.

“weight loss maintenance” and “strategy” identified 15 papers.

“weight loss maintenance” and “qualitative study” identified 8 papers.

“weight loss maintenance” and “cohort study” identified 5 papers.

#### PsycINFO

PsycINFO was searched from 1990 to the end of March 2020 using the terms below in three separate searches (“weight loss maintenance’’, “experience”, “behaviour”, “strategy”, “qualitative study” and “cohort study”). The searches were limited to “human” and “English language”.

“weight loss maintenance” and “behaviour” identified 28 papers.

“weight loss maintenance” and “experience” identified 26 papers.

“weight loss maintenance” and “strategy” identified 19 papers.

“weight loss maintenance” and “qualitative study” identified 36 papers.

“weight loss maintenance” and “cohort study” identified 3 papers.

Berry picking was also utilized to find related studies by entering some of the selected titles into Google Scholar to identify additional studies. Citations from reference lists were also evaluated for suitability.

## Appendix

**Table AI.** Evidence underpinning key concepts

Synthesis concept	Themes and sub-themes	Supporting study data	Supporting studies
Continuous Monitoring	Self-Monitoring: Calorie/portion tracking Meal Planning Home cooking Structured Plans Regular weighing Visual reminders Pre-planning for events Clear relapse protocols	Self-Monitoring tools utilized by WLMs: Calorie tracking, meal planning, meal prepping for work, decreasing portion sizes, weighing food, increasing vegetables and fruits, reducing alcohol intake, not ordering take out, home cooking, drinking more water/coffee/tea/diet drinks, avoiding junk food, activity monitors, tracking apps, regular weighing, structured programmes, eating low carb/low fat/high protein, skipping meals, compensation, coping strategies for high risk situations, food diaries, reading labels, reformulating recipes, purchasing low-calorie foods, compensation with exercise, goal adjustment, before-and-after pictures, planned cheat meals, distractions from eating e.g., more time outside, healthier menu options, healthy snacks, visual meal plans in kitchen, relapse protocols, infrequent, planned food shopping to limit exposure, replacing large plates, mindful eating, meal replacements, clothes fit as cue, establish routines, remove temptations, savouring treats, creation of emotional regulation strategies, continuous reading, recommending healthier restaurants for social events, pre-assessing menus for outings, swapping starches for vegetables	Kwasnicka et al. (2019), Sarlio-Lähteenkorva (2000), Sarlio-Lähteenkorva (2000), Natvik et al. (2019), Karfopoulou et al. (2013), Pedersen et al. (2018), Metzgar et al. (2015), Reilly et al. (2015), Kruseman et al. (2017), Cleo et al. (2018), Carrard and Kruseman (2016), Epiphaniou and Ogden (2010),, Barnes et al. (2007), McKee et al. (2013), Natvik et al. (2018), Ingels and Zizzi (2018), Natvik et al. (2018)
	External Monitoring: Weight support groups HCP feedback Online support Peer feedback	External monitoring utilized by WLMs: Weight maintenance groups, weight loss clubs (e.g., Weight Watchers), nutrition education about portion control, healthy eating, recipes and energy balance, mandatory reporting to HCPs, external weigh-ins, compliance assessments, external adjustment of goals, tailored, continuous feedback, healthy living support groups, support from colleagues and managers at work, support from local physician, online, on-going support from HCPs, support from friends and family members, regular educational group meetings, structured support, external encouragement, psychological support, researcher support, programme/study feeling of accountability	Sarlio-Lähteenkorva (2000), Karfopoulou et al. (2013), Metzgar et al. (2015), Cleo et al. (2018), Natvik et al. (2019), Metzgar et al. (2015), Reilly et al. (2015), Cleo et al. (2018), McKee et al. (2013), Natvik et al. (2018)
Motivation	Intrinsic motivators: Improving health Better self-image Stronger self-confidence Reinvention Better social opportunities (relationships, work)	Intrinsic motivators of WLMs: health, fitness, self-image, self-confidence, clothing size/slimness/vanity, new identity, liberation, relationship opportunities, quality of life, being in control, self-empowerment, normalcy, energy, sense of purpose, self-responsibility, mental health, increased self-efficacy, new skills, reinvention, new life, compliments	Kwasnicka et al. (2019), Sarlio-Lähteenkorva (2000), Natvik et al. (2019), Karfopoulou et al. (2013), Pedersen et al. (2018), Reilly et al. (2015), Kruseman et al. (2017), Carrard and Kruseman (2016), Epiphaniou and Ogden (2010), McKee et al. (2013), Natvik et al. (2018), Metzgar et al. (2015)
	Extrinsic motivators: Social standing/acceptance Decreased stigmatization Sense of belonging Career opportunities Being a role model	Extrinsic motivators of WLMs: social acceptance, social standing, career opportunities, decreased stigmatization, being a role model, impressing WL group peers, new identity, social respect, solidarity and sense of belonging from weight loss group, changing careers to help others lose weight	Sarlio-Lähteenkorva (2000) Karfopoulou et al. (2013), Carrard and Kruseman (2016), Epiphaniou and Ogden (2010), Barnes et al. (2007), Barnes et al. (2007), Natvik et al. (2018)
Goal Setting	Self- defined, personalized goals: Clear activity goals Mapped out eating goals Highly personalized goals Continuously reassessed goals Prioritization of goals Ongoing adjustment of goals	Self-defined goal setting by WLMs: Activity goals (e.g., gym visits, walking, cycling), eating goals (e.g., calories per day/week, macronutrients, portions), reduced alcohol intake, reduced snacking, increased vegetable and fruit intake, reflecting and continuously reassessing goals, refining and renewing goals, setting realistic goals, having realistic expectations, personalized goals, no “one-size-fits-all”, tailored approach, setting non-temporary goals, weight loss goals, lifestyle change goals, mutually facilitative goals (e.g., exercise leading to weight loss), prioritizing goals adequately	Kwasnicka et al. (2019), Natvik et al. (2019), Reilly et al. (2015), Kruseman et al. (2017), McKee et al. (2013), Natvik et al. (2018)
	Externally defined goals: Sports events HCP defined goals Weight loss club goals	Externally defined goal setting for WLMs: Cycling races, marathons, triathlons, HCP defined goals, HCPs continuously adjusting goals, on-going evaluation and assessment of goals, provision of external, neutral perspective on progress, utilization of pre-defined goal framework, programme defined goals (e.g., Weight Watchers goals)	Natvik et al. (2019), Reilly et al. (2015), McKee et al. (2013)
Enduring Challenges	Intrinsic challenges: Stress Lack of time Emotional eating Pregnancy/Illness/Injury Social isolation Overreliance on support system Lack of structure Unforeseen life events (death, divorce) Trigger foods	Intrinsic challenges faced by WLMs: stress, boredom, tiredness, lack of time, unhealthy food choices, sedentary behaviours, feelings of isolation, fear or regain, exhaustion, feeling disconnected from ‘former‘ self, lack of self-belief, inability to manage emotions, using foods to manage emotions, lack of awareness of emotional impact on WLM, weight cycling, appetite changes, psychological changes, feeling of deprivation, eating for pleasure, frustration over weight loss, urges, unforeseen circumstances, low impulse control with certain foods, lack of cooking skills, distorted sense of hunger, lack of accountability, feeling guilty for “taking time for themselves”, lack of willpower, lack of exercise, feeling guilty, retuning to “real” life, lack of focus, illness, injury, lifelong battle/effort, binge eating bouts, secret eating, pregnancy related weight gain, feeling out of control, frustration, lack of structure, low self-esteem, not prioritizing exercise, hairstyle concerns related to physical activity, injuries, feelings of disgust (e.g., in photographs), feeling powerless, loneliness, perceived unfairness, feeling restricted, social isolation, comfort eating, dichotomous thinking (all or nothing), disinhibited eating, loss of restraint, unforeseen life events, exercise increasing appetite, balancing responsibilities, overreliance on support system (e.g., HCPs, slimming clubs), exhaustion, constant fear of relapse	Kwasnicka et al. (2019), Sarlio-Lähteenkorva (2000), Ingels and Zizzi (2018), Karfopoulou et al. (2013), Pedersen et al. (2018), Pedersen et al. (2018), Metzgar et al. (2015), Reilly et al. (2015), Kruseman et al. (2017), Epiphaniou and Ogden (2010),, Barnes et al. (2007), McKee et al. (2013), Natvik et al. (2018)
	Extrinsic challenges: Work/personal stress Holidays Celebrations Relationship changes Obesogenic food environment Unsupportive peers Cultural norms	Extrinsic challenges faced by WLMs: Stress at work (e.g., tough meetings), house-renovations, traffic jams, unhealthy food option availability, being outside of the normal environment e.g., travelling, visiting family, holidays, cultural pressure to eat, social occasions e.g., celebrations, not eating seen as having bad manners, accusations of narrow mindedness, becoming disconnected, switching jobs, becoming a parent, relationship changes, reliance on others for emotional regulation, seasonal changes, temptations (sweets/desserts, calorie dense foods) in home, going out with friends, external cues to eat, food shopping, cooking for the household, not in charge of food preparation in the household, continuously declining foods at parties, family members criticizing results, conflict with household because of new food preferences, 24/7 food availability, snide remarks regarding food choices at restaurants, social gatherings and family meal times, work gatherings, unintentional sabotage, lack of family time, open discrimination, balancing responsibilities, cultural norms, finding communities with same lifestyle/new values	Kwasnicka et al. (2019), Sarlio-Lähteenkorva (2000), Ingels and Zizzi (2018), Karfopoulou et al. (2013), Pedersen et al. (2018), Metzgar et al. (2015), Reilly et al. (2015), Epiphaniou and Ogden (2010), Barnes et al. (2007), Natvik et al. (2018)
Overall Experience	Encouraging experiences: Feeling liberated Sense of Purpose New Identity Reinvention Compliments New Community Opportunities	Encouraging experiences with WLMs: new identity, liberation, relationship opportunities, self-empowerment, sense of purpose, self-responsibility, increased self-efficacy, new skills, reinvention, new life, compliments, social acceptance, social standing, career opportunities, decreased stigmatization, being a role model, impressing WL group peers, new identity, social respect, solidarity and sense of belonging from weight loss group, changing careers to help others lose weight	Epiphaniou and Ogden (2010), Natvik et al. (2019), McKee et al. (2013), Natvik et al. (2018), Kwasnicka et al. (2019), Sarlio-Lähteenkorva (2000)
	Discouraging experiences: Fear of Regain Loneliness Criticism Frustration Exclusion Isolation Feeling deprived	Discouraging experiences with WLMs: boredom, feelings of isolation, fear or regain, exhaustion, feeling disconnected from ‘former‘ self, lack of self-belief, inability to manage emotions, weight cycling, psychological changes, feeling of deprivation, frustration over weight loss, urges, unforeseen circumstances, lack of accountability, feeling guilty for “taking time for themselves”, lifelong battle/effort, feeling out of control, feeling guilty, frustration, low self-esteem, feelings of disgust (e.g., in photographs), feeling powerless, loneliness, perceived unfairness, feeling restricted, social isolation, overreliance on support system (e.g., HCPs, slimming clubs), exhaustion, constant fear of relapse, cultural pressure to eat, not eating seen as having bad manners, accusations of narrow mindedness, becoming disconnected, reliance on others for emotional regulation, family members criticizing results, conflict with household because of new food preferences, snide remarks regarding food choices at restaurants, social gatherings and family meal times, open discrimination	Sarlio-Lähteenkorva (2000), Natvik et al. (2019), Reilly et al. (2015), Kruseman et al. (2017), Kwasnicka et al. (2019), Metzgar et al. (2015), Pedersen et al. (2018), Karfopoulou et al. (2013), Natvik et al. (2018)
